# Trends in age- and sex-adjusted body mass index and the prevalence of malnutrition in children with cancer over 42 months after diagnosis: a single-center cohort study

**DOI:** 10.1007/s00431-019-03482-w

**Published:** 2019-10-28

**Authors:** Henri Aarnivala, Tytti Pokka, Riina Soininen, Merja Möttönen, Arja Harila-Saari, Riitta Niinimäki

**Affiliations:** 1 Department of Children and Adolescents, Oulu University Hospital and PEDEGO Research Unit, University of Oulu, Kajaanintie 52, 90220 Oulu, Finland; 2 Department of Women’s and Children’s Health, Uppsala University, Uppsala, Sweden

**Keywords:** Body mass index, Cancer, Chemotherapy, Child, ISO-BMI, Malnutrition

## Abstract

The adequate nutritional status of pediatric cancer patients is particularly important to enable them to cope with the demands of the disease and its treatment and to maintain normal growth. Malnutrition and obesity have both been associated with reduced survival and increased drug toxicity. We investigated trends in the age- and sex-adjusted body mass index (ISO-BMI) and the prevalence of malnutrition in a Finnish cohort of 139 consecutive children receiving chemotherapy for cancer, with a follow-up period of 42 months after diagnosis. In total, 28% (39/139) of the patients experienced malnutrition (ISO-BMI < 17 or > 10% weight loss), and 12% (16/139) had a nasogastric tube or underwent gastrostomy. Patients with acute or chronic myeloid leukemia (5/10), central nervous system (CNS) tumors (5/13), or solid tumors (13/31) most frequently suffered from malnutrition. There was a significant increase in the ISO-BMI of patients with acute lymphoblastic leukemia (ALL) (+ 2.1 kg/m^2^) and lymphomas (+ 2.4 kg/m^2^) during the first 6 months, and the ISO-BMI of patients with ALL remained higher at 42 months compared to baseline (+ 1.9 kg/m^2^).

*Conclusion*: The cumulative incidence of malnutrition in Finnish pediatric cancer patients is comparable to that reported in other populations. The nutritional status of patients with acute myeloid leukemia, CNS tumors, or solid tumors should be monitored with extra care to facilitate early intervention in the case of impending malnutrition.

**Table:** 

**What is known:** • *Both malnutrition and obesity are associated with reduced survival and increased drug toxicity in pediatric cancer patients.*	
**What is new:** • *Overall, 28 % of Finnish children receiving chemotherapy for cancer suffer from malnutrition during the first 42 months following the initial cancer diagnosis. * • *ISO-BMI curves from initial diagnosis to 42 months after diagnosis are provided for patients with different types of cancer.*

**What is known:**

• *Both malnutrition and obesity are associated with reduced survival and increased drug toxicity in pediatric cancer patients.*

**What is new:**

• *Overall, 28 % of Finnish children receiving chemotherapy for cancer suffer from malnutrition during the first 42 months following the initial cancer diagnosis. *

• *ISO-BMI curves from initial diagnosis to 42 months after diagnosis are provided for patients with different types of cancer.*

## Introduction

The prognosis of childhood cancer has improved significantly over the past 40 years, with 5-year survival now exceeding 80% [25]. However, almost 20% of pediatric cancer patients still die due to the disease itself or to the toxic effects of the treatment. Both the treatment and the disease influence the nutritional status of children with cancer. Adequate nutrition is important for children’s growth, development, and well-being [5], and in childhood cancer, it is particularly important to enable children to cope with the demands of the disease and its treatment and to maintain normal growth [27]. In pediatric cancer patients, malnutrition has been associated with reduced survival, increased risk of toxicity, decreased treatment tolerance, and higher risk of infection [7, 17, 20, 22]. Furthermore, obesity has been linked to reduced survival and increased toxicity-related mortality [17].

The prevalence of malnutrition and obesity varies depending on the type of cancer [8, 10, 16, 17]. According to the literature, the prevalence of malnutrition is up to 10% in leukemia, 20–50% in neuroblastoma, and up to 30% in other malignancies [6]. Lower prevalence rates have been reported in children with acute lymphoblastic leukemia (ALL), lymphomas, non-metastatic local tumors, or cancer in remission during maintenance therapy [30]. In two retrospective studies of pediatric patients with ALL, obesity at diagnosis increased the risk of relapse and decreased event-free survival [8, 14]. In patients with ALL, malnourishment has been associated with increased relapse rates and reduced survival in developing countries [18, 20]. In patients with acute myeloid leukemia (AML), both obesity and being underweight at the time of diagnosis have been shown to reduce survival and increase treatment-related mortality compared to healthy-weight patients [17].

The underlying etiology behind both malnutrition and obesity in patients with childhood cancer is multifactorial. Patients with advanced-stage disease and those receiving intensive treatment have the highest risk of malnutrition [6]. Treatment modalities reportedly increasing the risk of malnutrition include radiation of the gastrointestinal tract, major abdominal surgery, bone marrow transplantation, and frequent chemotherapy in the absence of corticosteroids [10]. Treatments increasing the risk of adiposity include corticosteroids, high-dose cranial/craniospinal radiotherapy, extensive brain surgery, and total-body or abdominal radiotherapy [10].

The primary aim of the present study was to investigate changes in the age- and sex-adjusted body mass index (ISO-BMI) and to determine and compare the incidence of malnutrition in children undergoing treatment for different types of cancer at a single center.

## Patients and methods

### Study cohort

**Fig. 1:**
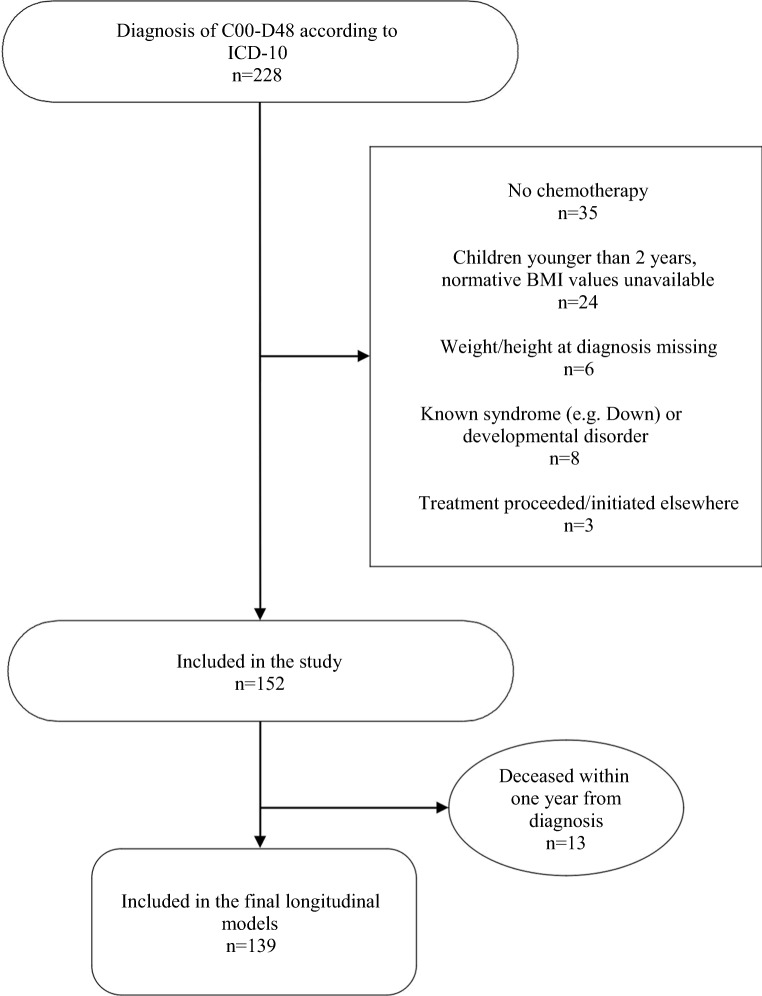
Study flow. ICD, International Classification of Diseases

We conducted a single-center, retrospective cohort study. All pediatric cancer patients who received a cancer diagnosis between January 2000 and December 2010 at the Oulu University Hospital, Oulu, Finland, were considered eligible for the study. We obtained all data retrospectively by reviewing the patients’ files, and there were no interventions involving the participants. Exclusion criteria were treatment without chemotherapy, age under 2 years at diagnosis (no normative BMI values available), known syndromes or apparent developmental disorders, cancer treatment administered at a hospital other than Oulu University Hospital, and missing weight and height measurements at the time of diagnosis. Additionally, subjects who died within 1 year after the initial cancer diagnosis were excluded from the final longitudinal analyses (Fig. 1). We categorized the subjects into five diagnostic groups: ALL, AML, or chronic myeloid leukemia (CML), lymphoma, solid tumor, and CNS tumor. The follow-up period lasted 42 months after the diagnosis or until the patient’s death, whichever occurred first.

### Weight and height measurements

We utilized the recently updated growth references for Finnish children using the BMI-for-age curves described for children aged 2–18 [21], dividing the weight status of the patients into four classes according to the ISO-BMI, with < 17 kg/m^2^ defined as underweight, 17–24.9 kg/m^2^ as healthy weight, 25–29.9 kg/m^2^ as overweight, and ≥ 30 kg/m^2^ as obese. The ISO-BMI values were derived from patients’ weight and height measurements at 6-month intervals throughout the follow-up period. Malnutrition during treatment was defined as being underweight in one or more measurements after the cancer diagnosis or losing more than 10% of weight [7, 27].

### Statistical analysis

We performed a linear mixed-model (LMM) analysis with a random intercept and a first-order autoregressive covariance structure for repeated measurements to assess changes in the BMI during the 42-month follow-up period [19]. We chose to use LMM analysis because it allows an unequal number of repetitions and uses the available values. We included a group-by-time interaction in the model to test for differences in means within and between the diagnostic groups at each time. We analyzed distribution differences between the categorical variables and the chi-square test and differences between continuous variables and Student’s *t* test. We used multiple logistic regression to calculate risk ratios [1]. We used the Kaplan–Meier method to assess survival time after diagnosis [15]. We performed all analyses using SPSS for Windows, version 22.0.0 (IBM Corp, Armonk, NY, USA) and drew the graphs with OriginPro 9.1.0 (OriginLab, Northampton, MA, USA). All results are expressed as *n* (%) or mean ± standard deviation (SD) unless stated otherwise.

## Results

### Study cohort

A total of 139 patients for whom growth data were available for at least 12 months after diagnosis were included in the study. Of these, 87 (63%) were male, and the mean age at diagnosis was 7.4 ± 4.3 (range 2–16) years. At diagnosis, 9% of these subjects were underweight, 71% were a healthy weight, 16% were overweight, and 4% were obese. There was no statistically significant difference in the mean ISO-BMI at diagnosis between the different diagnostic groups (Table 1). In the AML/CML group, all three patients with CML received a stem cell transplantation. Of all the patients, 83% (115/139) remained alive until the end of the follow-up.

**Table 1:** Descriptive statistics for the 139 patients included in the final longitudinal analysis divided by cancer type. Values expressed as mean ± SD or *n* (%)

	ALL (*n* = 68)		AML/CML (*n* = 10)		Lymphomas (*n* = 17)		Solid tumors (*n* = 31)		CNS tumors (*n* = 13)	
ISO-BMI at diagnosis	21.6	± 3.9	21.1	± 3.8	22.5	± 3.7	21.7	± 5.0	20.5	± 3.3
ISO-BMI at 42 months	23.3	± 4.4	22.8	± 6.1	24.6	± 3.9	22.1	± 4.0	20.7	± 3.4
Radiation therapy	11	(16)	6	(60)	2	(12)	19	(61)	10	(77)
Corticosteroids as part of cancer therapy	68	(100)	0	(0)	17	(100)	0	(0)	0	(0)
Stem cell transplant	10	(15)	6	(60)	0	(0)	3	(10)	0	(0)
Malnutrition^1^ between 6 to 42 months	14	(21)	5	(50)	2	(12)	13	(42)	5	(38)
Nasogastric tube	3	(4)	3	(30)	0	(0)	4	(13)	2	(15)
Gastrostomy tube	1	(1)	2	(20)	0	(0)	3	(10)	2	(15)
Nasogastric or gastrostomy tube	3	(4)	4	(40)	0	(0)	6	(19)	3	(23)
Alive at 42 months	61	(90)	8	(80)	16	(94)	22	(71)	10	(77)

^1^ISO-BMI < 17 kg/m^2^ or > 10% weight loss

**Fig. 2:**
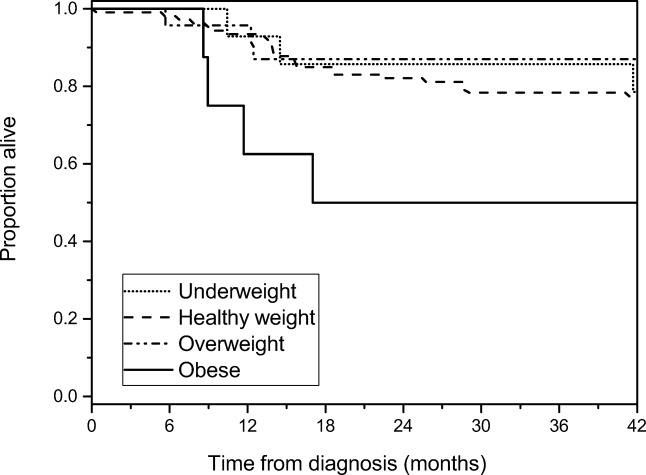
Kaplan–Meier survival plot of the 152 patients meeting the inclusion criteria for the study according to weight status at diagnosis

There were initially 152 patients meeting the inclusion criteria, but 13 died within 12 months of diagnosis, precluding the analysis of repeated follow-up ISO-BMI measurements in these patients. Therefore, they were excluded from the final longitudinal models. Highly malignant CNS tumors were most often (9/13) the cause of early death, but refractory hematological malignancies (2/13), metastatic alveolar rhabdomyosarcoma (1/13), and metastatic melanoma (1/13) were also represented. The mean time from diagnosis to death was 6 ± 2 months in this subgroup. Univariate analyses showed that obesity at diagnosis was associated with an increased risk of death within 12 months of diagnosis (crude risk ratio [RR] 5.4; 95% CI, 1.8–17.5; *p* < 0.001) and death within 42 months (crude RR 2.2; 95% CI, 1.1–4.7; *p* < 0.05). When adjusted for cancer type, patient age, and gender in a logistic regression model, the risk of death within 12 months remained statistically significantly higher (adjusted RR 4.6; 95% CI, 1.1–17.9; *p* < 0.05), but death within 42 months did not (adjusted RR 1.9; 95% CI, 0.7–9.0; *p* = 0.24). There was no association between being underweight at diagnosis and death within 12 months (crude RR 0.8; 95% CI, 0.1–5.8; *p* = 0.84) or death within 42 months (crude RR 0.9; 95% CI, 0.3–2.5; *p* = 0.82). Survival in relation to weight status at diagnosis is illustrated in Fig. 2.

### Trends in ISO-BMI

**Fig. 3:**
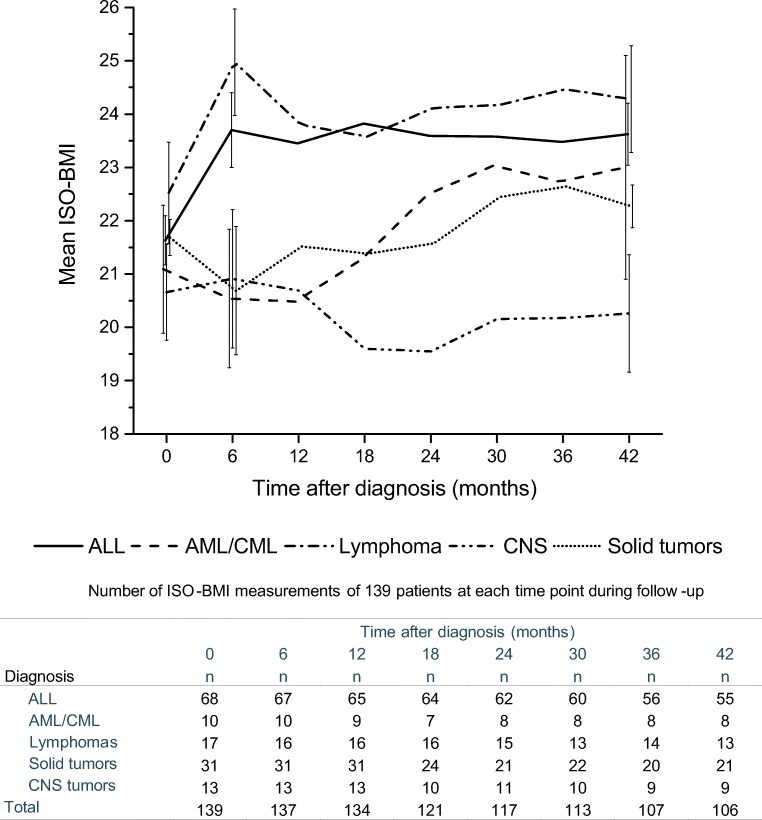
The course of mean age- and sex-adjusted body mass index (ISO-BMI) in the different diagnostic groups over the first 42 months after cancer diagnosis. Error bars at the 0-, 6-, and 42-month time points indicate ± one *standard error*

The course of ISO-BMI clearly differed between the diagnostic groups during the 42-month follow-up period (Fig. 3). According to the LMM analysis, ISO-BMI increased to a statistically significant extent compared to baseline in patients with ALL (+ 2.1 kg/m^2^; 95% CI, 1.5–2.7; *p* < 0.001) and in patients with lymphomas (+ 2.4 kg/m^2^; 95% CI, 1.2–3.7; *p* < 0.001) during the first 6 months following the initial diagnosis. The ISO-BMI of patients with solid tumors decreased during the first 6 months (− 1.0 kg/m^2^; 95% CI, − 1.9–− 0.1; *p* < 0.05), gradually increasing thereafter. Patients with AML/CML or CNS tumors showed no constant trends in the course of ISO-BMI, and at 42 months there was no significant difference in the ISO-BMI of patients with solid tumors, AML/CML, or CNS tumors compared to their ISO-BMI at diagnosis. The ISO-BMI remained significantly higher at 42 months compared to the ISO-BMI at diagnosis in patients with ALL (+ 1.9 kg/m^2^; 95% CI, 1.0–2.8; *p* < 0.001) but not in patients with lymphomas (+ 1.1 kg/m^2^; 95% CI, − 0.7–3.0; *p* = 0.24).

The weight gain of patients with ALL or lymphomas in the first 6 months after diagnosis differed significantly from the more stable course observed in patients in the three remaining diagnostic groups (difference in mean change in ISO-BMI: (ALL vs. CNS tumors, AML/CML, and solid tumors: 2.0–3.0 kg/m^2^; 95% CI, 0.3–4.6; *p* < 0.01), (lymphomas vs. CNS tumors, AML/CML, and solid tumors: 2.4–3.4 kg/m^2^; 95% CI, 0.3–5.3; *p* < 0.01)). However, by 42 months, the differences had waned and only the increase in the ISO-BMI of patients with ALL retained statistical significance compared to patients with CNS tumors (2.4 kg/m^2^; 95% CI, 0.3–4.5; *p* < 0.05) and patients with solid tumors (1.6 kg/m^2^; 95% CI, 0.1–3.1; *p* < 0.05). Neither receiving radiation therapy nor receiving a stem cell transplant had an effect on the course of ISO-BMI in the LMM analysis.

### Malnutrition

The incidence of malnutrition in the 42 months after the initial cancer diagnosis is summarized in Table 1. Of the patients, 28% (39/139) suffered from malnutrition during cancer treatment. Malnutrition was more common in patients with AML/CML, solid tumors, or CNS tumors compared to patients with ALL or lymphomas (RR 2.3; 95% CI, 1.3–3.9; *p* < 0.001). Nasogastric or gastrostomy tubes were placed in 12% (16/139) of the patients for nutritional reasons (Table 1). Patients with AML/CML, solid tumors, or CNS tumors were more likely to receive tube feeding than patients with ALL or lymphomas (RR 6.8; 95% CI, 2.0–22.8; *p* < 0.001). All three ALL patients receiving tube feeding were treated with intensified chemotherapy due to high-risk disease. Data on the time of initiation and duration of tube feeding were available for 14/16 and 13/16 patients, respectively. The mean time from diagnosis to tube placement was 7.0 ± 6.9 (range 0–21) months, and the duration of tube feeding was 5.5 ± 3.4 (range 0.6–12) months, excluding one patient who could not be weaned from tube feeding for neurological reasons. Eight of the 14 patients met the criteria for malnutrition at the time of feeding tube placement. The decrease in ISO-BMI from diagnosis until the initiation of tube feeding stood at a median of − 3.5 kg/m^2^ (interquartile range Q1–Q3: − 4–− 1.4 ), and by the next 6-month measurement following the cessation of tube feeding, ISO-BMI had increased by a median of 1.6 kg/m^2^ (interquartile range Q1–Q3: 1.0–3.7).

## Discussion

This observational, single-center, retrospective cohort study provides insight into the course of ISO-BMI and the prevalence of malnutrition in the 42 months after the initial cancer diagnosis in pediatric patients receiving chemotherapy for different types of cancer. The course of ISO-BMI was dependent on cancer type. Patients with ALL or lymphomas were likely to gain weight during the follow-up period, whereas the course of ISO-BMI showed no obvious or uniform trend in patients with AML/CML, solid tumors, or CNS tumors.

Several previous studies have reported patients with ALL being at risk of gaining weight during cancer treatment [4, 13, 28], which is consistent with the results of the present study. In our cohort, patients with ALL gained weight during the first 6 months after the cancer diagnosis, whereafter their ISO-BMI consistently remained at the level it had increased to, both during the maintenance phase and following treatment cessation until the end of the follow-up. Previous studies report a similar pattern of weight changes in patients with ALL during therapy [9, 13]. These studies generally attributed the changes to corticosteroid exposure and related insulin resistance, as the patients gained weight during the first month of therapy, after which their weight returned to baseline but increased again during maintenance therapy. However, longer follow-up studies on children with a history of ALL or lymphoma have shown that both diseases increase the risk of obesity for years after the cessation of cancer treatment [2, 29], which is in line with the present results. The prolonged risk of weight gain suggests that factors other than corticosteroids also contribute to the problem. For example, pediatric patients previously treated for ALL or lymphoma reportedly have a higher craving for fast food and more frequent food cravings [24]. Hypothalamic–pituitary axis dysfunction is the commonly proposed pathomechanism behind the elevated risk of weight gain, a problem that may be caused by the tumor itself, treatment [12], or reduced physical activity [3]. ALL patients also are at a higher risk of developing metabolic syndrome, which again is amplified by physical inactivity both during and after therapy. Recently, several physical training intervention protocols have become available for children with ALL, and promising results regarding both obesity and metabolic alterations are being published [26]. Hence, nutritional counseling should be provided and physical activity promoted both during and after the cessation of ALL treatment in order to decrease subsequent long-term health risks.

In our cohort, the cumulative incidence of malnutrition was 28% in pediatric cancer patients in the 42 months following cancer diagnosis, with patients treated for AML/CML, solid tumors, or CNS tumors having the highest rates of malnutrition. The risk of having a nasogastric or gastrostomy tube placed was also considerably higher in the aforementioned disease groups than in patients with ALL or lymphomas. However, there was no association between receiving radiotherapy or a stem cell transplant and the course of ISO-BMI or the risk of malnutrition. On the other hand, the higher incidence of malnutrition and the lower mean ISO-BMI in the CNS tumor group may partly be explained by tumor- or therapy-related hypopituitarism. The incidence rates of malnutrition described are in the range of those reported in the literature, while even higher rates of malnutrition have been reported by, for example, Zimmermann et al., who reported a cumulative incidence of malnutrition as high as 47% in children and young adults with cancer, with the risk of malnutrition being the highest in patients with medulloblastomas [30]. The factors implicated in the development of malnutrition in childhood cancer include decreased energy intake and malabsorption; altered protein, lipid, and carbohydrate metabolism; an increased metabolic rate; and inflammation [6, 11, 22].

In our study, patients who were obese at the time of diagnosis appeared to have a higher risk of death within 12 months of diagnosis, even when adjusted for disease type. However, the death of only one of the three obese patients who died within 12 months was clearly attributable to drug toxicity (lymphoma, stubborn leukopenia leading to fatal infection). Furthermore, as our cohort was relatively small and included patients with a wide range of diagnoses, it was not feasible to further divide the patients by tumor location, treatment regimen used, etc. Therefore, we were unable to further adjust the statistical analyses for these factors. Consequently, we are cautious about making a correlation between nutritional status and survival based on the present data.

The adequate nutritional status of pediatric cancer patients is important to enable them to cope with the demands of the disease and its treatment and to maintain normal growth [27]. Therefore, it is important to carefully monitor the weight status of pediatric patients with cancer and to implement nutritional interventions when necessary. Malnutrition during cancer treatment was very common in our center, even though patients’ weight and height were generally measured at monthly intervals, the multidisciplinary treatment team included a dietitian, and both enteral and parenteral nutrition were used. Furthermore, it was more an exception rather than the rule to have a nasogastric or gastrostomy tube placed *before* the patients’ ISO-BMI had decreased below 17 kg/m^2^ or the patient had lost > 10 % of their weight. These facts highlight the need for earlier interventions and universal guidelines for when and how to commence enteral feeding by nasogastric or gastrostomy tubes in pediatric cancer patients. A study by Schmitt et al. investigated the efficacy of preventive gastrostomy feeding, and the authors reported that osteosarcoma patients who underwent percutaneous endoscopic gastrostomy at the time of diagnosis and received continuous enteral tube feeding throughout chemotherapy had a higher rate of survival [23]. Whether such measures are justified in clinical practice and in which types of cancer are questions for future research to answer.

The strengths of the present study include its single-center design, the systematic registration of weight and height measurements, and including all patients receiving chemotherapy for whatever type of cancer over 10 years, thus well representing the original population. However, having to exclude children under two at diagnosis due to a lack of normative weight and height data was a limitation. The relatively small sample size was another limitation, precluding reliable estimation of the impact of weight status on survival. Another limitation was the exclusion of patients who died within 12 months of diagnosis from the longitudinal analyses, which may have introduced bias into the LMM results for patients with CNS tumors in particular, but on the other hand, very unlikely on the essential results of the study. Finally, the definition of malnutrition is always somewhat arbitrary, and although we used a definition similar to those used in previous studies [7, 27], there is still an obvious need for uniform diagnostic criteria for malnutrition in pediatric cancer patients to enable a reliable comparison of results from different studies and populations.

## Conclusions

Malnutrition during the treatment of malignancy is very common in pediatric patients, even when a variety of nutritional interventions are readily available. Patients with ALL or lymphomas are at a lower risk for malnutrition but at a higher risk of gaining weight. The importance of nutrition, the risk of malnutrition and/or obesity, and the potential interventions needed in the case of malnutrition should be discussed in the early stages of treatment with both the child and the child’s guardians to facilitate early recognition and intervention. An obvious need for both diagnostic criteria for malnutrition and guidelines for its prevention and treatment in the pediatric cancer patient population persists.

## Abbreviations

ALL: Acute lymphoblastic leukemia
AML: Acute myeloid leukemia
BMI: Body mass index
CI: Confidence interval
CML: Chronic myeloid leukemia
CNS: Central nervous system
ISO-BMI: Age- and sex-adjusted body mass index
LMM: Linear mixed model
RR: Risk ratio
SD: Standard deviation
